# Supplementary material for: Keratin Expression in Podocytopathies, ANCA-Associated Vasculitis and IgA Nephropathy
Source: Int J Mol Sci. 2024 Feb 2;25(3):1805. doi: 10.3390/ijms25031805 (PMC10855225; doi:10.3390/ijms25031805)
Supplement: Supplementary file 1 [file ijms-25-01805-s001.zip › ijms-2800861-supplementary.pdf]

Supplementary Table S1. List of antibodies used for immunohistochemistry.

**Target**	**Clone/Label**	**Host**	**Supplier**
K7	OV-TL12/30	mouse	Dako
K8	EP1628Y	rabbit	Thermo scientific
K18	RCK 106	mouse	Progen
K19	KS19.1	mouse	Thermo scientific
Mouse IgG	biotin	sheep	Vector
Rabbit IgG	biotin	goat	Vector
